# Rust and redemption: iron–sulfur clusters and oxygen in human disease and health

**DOI:** 10.1093/mtomcs/mfaf022

**Published:** 2025-07-02

**Authors:** Shany Egozi, Tslil Ast

**Affiliations:** Department of Biomolecular Sciences, Weizmann Institute of Science, Rehovot, Israel; Department of Biomolecular Sciences, Weizmann Institute of Science, Rehovot, Israel

## Abstract

Iron–sulfur (Fe–S) clusters are ancient and versatile cofactors that drive essential cellular functions, from electron transport to enzyme catalysis. Their intrinsic sensitivity to oxidation has shaped the evolution of specialized Fe–S cluster biosynthetic and protective mechanisms. Recent findings highlight how human Fe–S-binding regulators exploit this cofactor's reactivity to sense iron and oxygen levels, translating environmental cues into appropriate homeostatic responses. Yet, the same redox sensitivity also renders Fe–S cluster proteins and biosynthesis particularly vulnerable to high oxygen tensions, contributing to pathological outcomes. In this minireview, we examine key discoveries illustrating how Fe–S clusters and oxygen intersect to influence both human health and disease. Finally, we discuss how identifying novel Fe–S targets and regulatory circuits may open innovative therapeutic avenues—harnessing oxygen itself as a strategic element in managing relevant disorders.

## Graphical Abstract

**Graphical Abstract:**
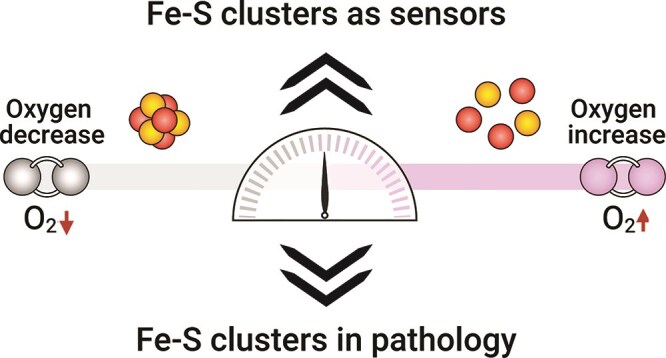

## Introduction—Fe–S clusters in the transition to an oxygen-rich world

Iron–sulfur (Fe–S) clusters are among the oldest known cofactors, thought to have played a critical role in the emergence of life on Earth [1]. As versatile catalysts for electron transfer and redox reactions, these inorganic cofactors likely underpinned fundamental processes in early biochemical pathways. Their ability to mediate energy conversion in the absence of oxygen suggests they were crucial in primitive metabolic networks [1].

Fe–S clusters can assemble spontaneously from Fe^2+^ and sulfide in the absence of oxygen [2], conditions reminiscent of the primordial oceans before Earth's oxygenation [3]. However, cells have developed sophisticated systems to actively assemble these essential cofactors. Comparative genomic analyses have revealed the conservation of Fe–S cluster biogenesis genes across all domains of life [4]. These systems possess structural and functional characteristics optimized for anaerobic conditions, supporting their early adaptation to a low-oxygen environment. Two distinct minimal Fe–S cluster biogenesis machineries have been found, likely already present in the last universal common ancestor, which possibly represent the origin of present-day systems [4]. This new data point to the emergence of Fe–S cluster biosynthesis machinery before the Great Oxygenation Event, contradicting the hypothesis that oxidative stress drove the formation of this system.

The rise in atmospheric oxygen following the Great Oxidation Event posed a challenge to Fe–S cluster biosynthesis and stability due to their labile nature [5]. First, the oxidation of Fe^2+^ to Fe^3+^ by oxygen causes it to precipitate or form insoluble complexes, limiting its bioavailability. Second, oxidation of an Fe–S cluster, either by molecular oxygen or by reactive oxygen species (ROS), destabilizes its structure and results in its disassembly. In addition to structural deterioration, oxidative modifications of Fe–S clusters impair their ability to participate in redox reactions and electron transport [6]. This disruption compromises the function of Fe–S dependent proteins, affecting key metabolic processes.

Cells have developed different parallel strategies to manage the effects of increased oxidative burden. Some of these strategies include shielding the cluster with protein scaffolds to protect it from the oxygen in the cell [5,7]. Another is the emergence of antioxidant enzymes, such as superoxide dismutase and catalase, that act to neutralize ROS [8]. Finally, in some instances, Fe–S clusters have been swapped for more stable cofactors, or alternative pathways have emerged [9]. However, this oxidative “Achilles heel” of Fe–S clusters has also been exploited in some contexts, leading to the emergence of redox-responsive proteins. Taken together, evolutionary pressures shaped the biosynthesis and protection of Fe–S clusters as life adapted to an oxygenated world.

## Physiological sensing by Fe–S clusters

Well-tuned to the cell's iron and oxygen status, Fe–S clusters serve as ideal indicators in environmental sensing systems. Examples of such systems are found throughout the Tree of Life. The bacterial RirA, which negatively regulates the transcription of iron homeostasis genes, contains a labile [4Fe-4S] cluster [10]. This cluster promotes DNA binding, ensuring that RirA is active only when iron is abundant, and the bacteria is growing in low oxygen tensions. In yeast, a conceptually similar transcriptional programme is carried out by the Fe–S cluster binding transcriptional factors Aft1, Aft2, and Yap5 [11,12].

In mammalian cells, there are at least four examples of iron and oxygen-response proteins, which hinge on Fe–S cluster sensors.

**Figure 1.**
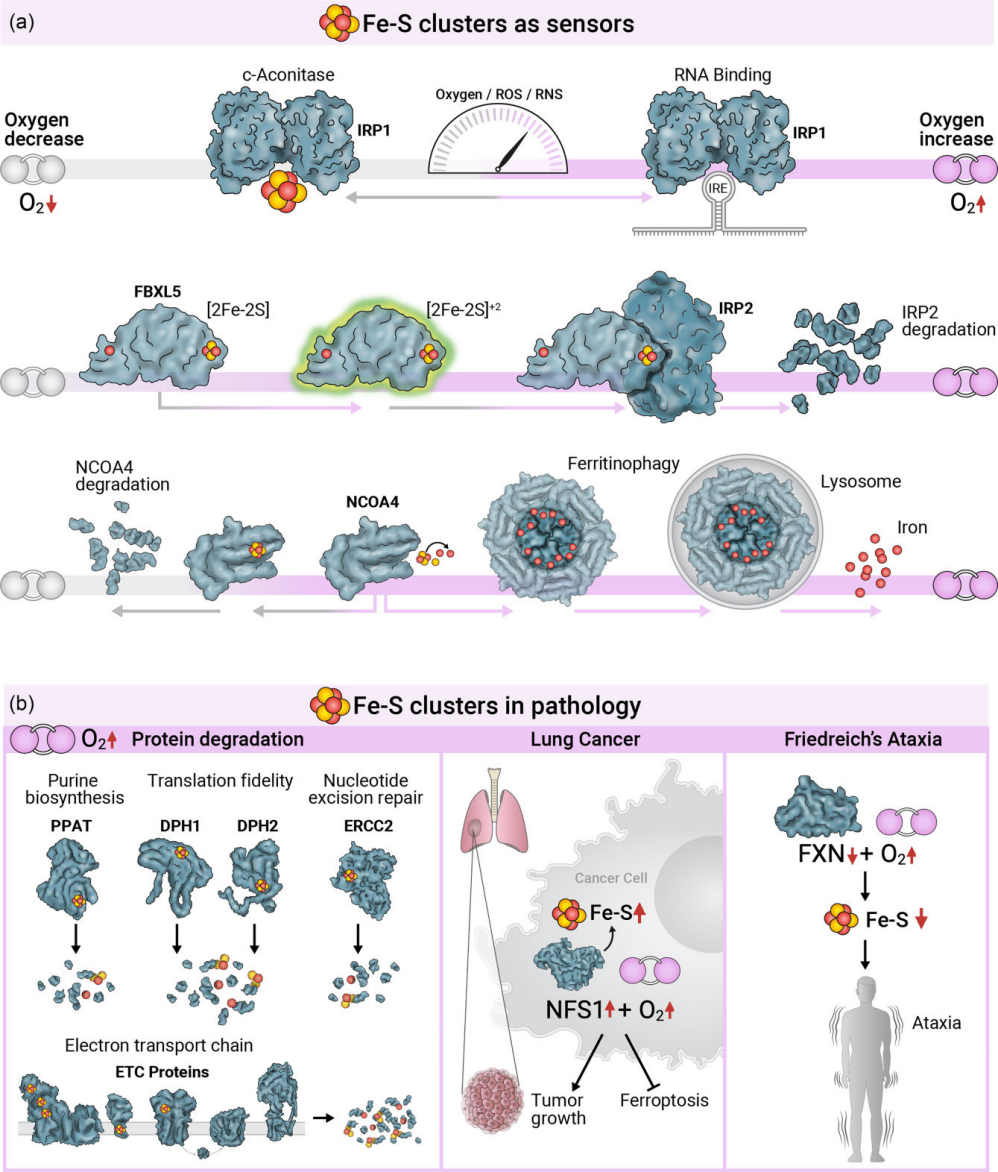
Fe–S clusters and oxygen shaping human health and disease. (a) Top-Oxygen tensions and ROS/RNS levels regulate IRP1 function. Under low oxygen tensions, IRP1 retains its [4Fe-4S] cluster and functions as a cytosolic aconitase. At higher oxygen tensions, the cluster degrades, shifting IRP1 to its RNA-binding role as an iron regulator. Middle-FBXL5 function is oxygen-dependent. Under high oxygen tensions, the [2Fe-2S] cluster in its LRR domain becomes redox-activated, enabling FBXL5 to recognize its target proteins, IRP1/2. This recognition leads to the ubiquitination and subsequent proteasomal degradation of the target proteins. Bottom- NCOA4 function is modulated by oxygen. At low oxygen tensions, NCOA4 acquires an Fe-S cluster, marking it for recognition and degradation via the proteasome. At high oxygen tensions, apo-NCOA4 targets ferritin to the phagolysosome, releasing free iron for cellular use via ferritinophagy. (b) Left- Hyperoxia induces the degradation of Fe–S cluster proteins, including PPAT, DPH1/2, ERCC2, and several proteins in ETC complexes I and II. This degradation disrupts the pathways of purine metabolism, diphthamide synthesis, nucleotide excision repair, and ETC, respectively. Middle- In lung cancer upregulation of the Fe-S cluster biosynthesis protein NFS1 inhibits ferroptosis in the face of high oxygen tensions, promoting cell proliferation and tumor growth. Right—in Friedreich's ataxia, characterized by reduced FXN protein levels, high oxygen tension accelerates ataxia symptom onset and worsens disease progression.

The first discovered sensor was iron response protein 1 (IRP1). IRP1 has two possible functions, dependent on its cluster binding state. The first is metabolic, namely, when bound to a [4Fe-4S] cluster, IRP1 acts as cytosolic aconitase, isomerizing citrate to isocitrate (Fig. 1a-Top) [13]. Alternatively, in its cluster-free state, IRP1 functions as a translational regulator, modulating transcripts involved in iron homeostasis [14]. In this apo-state, IRP1 engages with short hairpin structures on its target mRNAs termed iron-responsive elements (IREs) (Fig. 1a-Top). Notably, bacterial aconitase activity has long been known to be modulated by ROS and reactive nitrogen species (RNS) due to the solvent-accessible nature of its cluster [15]. This sensitivity extends to mammalian aconitase, where RNS bursts created in activated macrophages have been shown to induce IRP1 RNA binding activity [16,17]. Conversely, hypoxia appears to blunt the ability of IRP1 to bind target IREs, possibly stabilizing the protein in the cluster-bound aconitase form [18]. Thus, both iron and oxygen levels act as modulators of IRP1 activity.

Cellular iron and oxygen responsive pathways are also regulated by dedicated protein degradation systems, which engage an Fe–S cluster. The E3 ubiquitin ligase FBXL5 acts as a key negative regulator of the iron response proteins 1 and 2 (IRP1 and IRP2), targeting them for degradation (Fig. 1a-Middle) [19,20]. FBXL5 function is dependent on two different co-factors: Its N-terminal hemerythrin domain binds a di-iron center, while a [2Fe-2S] cluster is found at its C-terminal leucine-rich repeat (LRR) domain. This LRR domain forms a primary binding site for IRP1/2, and thus the presence of the Fe–S cluster is essential for IRP1/2 recognition [21]. Notably, FBXL5’s [2Fe-2S] cluster is redox active, modulating IRP1/2 binding as a factor of oxygen tensions (Fig. 1a-Middle). Specifically, oxygen shifts the cluster to its [2Fe-2S]^+2^ state, which facilitates target engagement. Similarly, under oxidative stress conditions, FBXL5 protein levels increase, and IRP2 degradation is amplified [22]. Further underlining its connection to oxygen sensing, FBXL5 was shown to engage the cytosolic Fe–S cluster assembly (CIA) machinery only under high oxygen tensions. This interaction promotes the ability of FBXL5 to degrade IRP1/2 [23]. It remains to be discovered whether the interaction between FBXL5 and the CIA machinery is purely regulatory or if FBXL5 receives its Fe–S cluster from this complex.

The release of iron from cellular storage depots has been recently shown to be controlled by an oxygen sensitive Fe–S cluster. This process, termed ferrintophagy, involves the lysosomal degradation of ferritin via selective autophagy. Ferrintophagy is orchestrated by the protein nuclear receptor coactivator 4 (NCOA4), a selective cargo receptor [24,25]. NCOA4 has been recently shown to bind an Fe-S cluster, rendering it responsive to cellular iron and oxygen levels (Fig. 1a-Bottom) [26,27]. The binding of the Fe–S cluster to NCOA4, which is favored under low oxygen and high-iron conditions, induces its recognition and ubiquitination by the E3 ubiquitin ligase HERC2. As a result, NCOA4 undergoes proteasomal degradation and ferritin accumulates, promoting iron storage within the cell. Conversely, under high oxygen and low-iron conditions, NCOA4 loses its cluster and shifts to the apo form (Fig. 1a-Bottom). This cluster-free version of NCOA4 targets ferritin to the phagolysosome, releasing free iron for cellular use [26,28]. In this manner, cellular iron levels are rapidly tuned to local oxygen fluctuations.

Finally, the outer mitochondrial membrane protein CISD1 (i.e. mitoNEET), which harbors a unique [2Fe-2S] cluster coordinated by three cysteines and one histidine, is emerging as a redox-sensitive regulator [29–33]. Although its precise function remains an area of active investigation, CISD1 has been proposed to donate its Fe–S cluster to cytosolic apo-proteins, such as IRP1[34]. Recent studies demonstrate that cluster transfer occurs only when CISD1’s [2Fe-2S] cluster is in the oxidized +2 state, highlighting a redox-gated mechanism [34–36]. Molecular oxygen promotes this oxidized state and, under acidic conditions (pH < 7), destabilizes the cluster [37,29]. Additionally, CISD1’s cluster can react with gasotransmitters, such as hydrogen sulfide and nitric oxide (NO), although oxygen competitively inhibits NO binding [37]. These properties suggest that CISD1 operates as an oxygen- and redox-sensitive switch.

## Disease at the crossroads of Fe–S clusters and oxygen

Excess oxygenation has long been recognized as toxic, first documented in animal models at the end of the 19th century [38,39]. Beyond the clinical setting of supplemental oxygen administration, tissue hyperoxygenation can arise in various pathological conditions. Indeed, multiple disorders disrupt normal tissue oxygen thresholds, including ischemia-reperfusion injury, obstructive sleep apnea, bronchopulmonary dysplasia, and mitochondrial diseases [40–41,42,43]. However, the molecular determinants of this toxicity have remained poorly defined in metazoa. A recent combination of proteomics and genetic screening has delineated four human pathways that are especially vulnerable to high oxygen tensions [44]. Purine metabolism, diphthamide synthesis, nucleotide excision repair, and the electron transport chain (ETC) activity were attenuated when cultured cells were grown at 80% O_2_. Underlying these blockades were highly labile Fe–S cluster proteins- PPAT, DPH1/2, ERCC2, and different complex I and II subunits in the ETC, respectively (Fig. 1b-Left). Excess oxygen is known to trigger the formation of ROS, such as superoxide. Indeed, in bacterial systems, it has been shown that [4Fe-4S] cluster binding dehydratases (e.g. enzymes in the branched-chain amino acid biosynthesis and the Krebs cycle) are particularly vulnerable to superoxide stress [45–46]. However, superoxide-neutralizing antioxidants were ineffective at mitigating hyperoxia damage to these four key metazoan pathways, suggesting a direct link to molecular oxygen [44]. Intriguingly, hyperoxia-induced damage was also shown to be self-propagating. The degradation of ETC proteins blunts mitochondrial oxygen consumption, further increasing cellular oxygen levels and oxidative damage. Thus, therapeutic strategies aimed at preserving Fe–S clusters may mitigate tissue injury in disorders linked to elevated oxygen.

Tissues are typified by distinct oxygenation “set-points”, ranging widely from the highly oxygenated lung to more oxygen-scarce organs such as the uterus and large intestine [47,48]. Systematic analyses of genes selectively essential for cell survival at high oxygen tensions have revealed a heightened dependence on the Fe–S cluster biosynthesis pathway [49,50]. This is further supported by the finding that lung tumors, particularly lung adenocarcinomas, elevate the production of Fe–S clusters [50]. Specifically, the cysteine desulfurase NFS1, essential for sulfur donation, is under positive selection and is prone to genomic amplification in lung adenocarcinomas (Fig. 1b-Middle). Alternatively, suppression of NFS1 under high-oxygen tensions sensitizes cells to ferroptosis, an iron-dependent form of cell death, which causes tumor growth arrest and the prevention of its metastasizing (Fig. 1b-Middle). Disruption of Fe–S cluster biosynthesis commonly results in cellular iron accumulation and increased oxidative burden. This potent combination triggers the Fenton reaction, producing highly reactive hydroxyl radicals and lipid peroxidation, the key insult underlying ferroptosis. For in-depth discussions of ferroptosis, readers are referred to Lee et al. (2023) and Dixon et al. (2024) [51,52]. Notably, depletion of NFS1 was well tolerated in more poorly oxygenated mammary or subcutaneous tumors [50]. This makes NFS1 suppression, or its downstream pathway, a plausible target for treating tumors in high-oxygen environments such as the lungs.

Finally, while Mendelian diseases that affect Fe–S cluster biogenesis are analyzed mostly in the context of their genetic deficiencies, it is possible that oxygen is a potent environmental factor contributing to their pathology. The most common of these diseases is Friedreich's ataxia (FRDA), which affects 1:50 000 individuals and is caused by mutations in the gene FXN. FRDA is a multisystemic disorder with symptoms such as ataxia, gait abnormalities, cardiomyopathy, vision impairment, scoliosis, and a heightened risk of diabetes [53–54,55]. FXN appears to function as an allosteric activator of Fe–S cluster biogenesis, facilitating persulfide formation on NFS1, and its transfer to the biogenesis scaffold- ISCU [56–57,58]. Highlighting a link to oxygen in FRDA, a study that examined yeast, human cells, and nematode models of the disease showed that hypoxia (1% O_2_) significantly improves all molecular symptoms of the disease (Fig. 1b-Right) [59]. FXN, but not other components of the core Fe–S cluster biogenesis machinery, becomes dispensable for viability in hypoxia. Interestingly, these effects are independent of hypoxia signalling pathways (i.e. HIF signaling) and could be recapitulated upon *in vitro* reconstitution assays. These observations extended to preclinical models of FRDA, ataxia was prevented in an FRDA mouse model housed in low oxygen (11% O_2_), and follow-up work showed that this oxygen tension could also reverse ataxia [60]. Furthermore, if the FRDA mice were exposed to 55% O_2_, symptoms of ataxia manifested early, indicating that high oxygen could worsen disease progression (Fig. 1b) [59]. Highlighting tissue divergence in FRDA pathology, the cardiomyopathy associated with FRDA was not improved in chronic continuous exposure to 11% O_2_. This research underscores the therapeutic potential of hypoxia or hypoxia-mimicking interventions, and the need for additional preclinical work on this gene: environment interaction.

## Summary

While the existence of Fe–S clusters within biological systems has been appreciated for over 8 decades, it is only recently that the physiological and pathological interplay of these inorganic cofactors with oxygen has been revealed. Thus, many questions remain unanswered in this field. As we lack the entire scope of human Fe–S cluster binding proteome, it is likely that the extent of oxidative-responsive pathways is currently underestimated. This knowledge may also help shed light both on the molecular underpinnings of Fe–S cluster biosynthesis diseases and on tissue hyper-oxygenation. This leads to another challenge in the matter—how should oxygen be incorporated into the treatment of patients who suffer from these disorders? Ultimately, a deeper understanding of the role of Fe–S clusters in an oxygen-rich world may not only contribute to our understanding of cellular metabolism but also pave the way for novel therapies and treatments.

## Data Availability

No new data were generated or analyzed in support of this research.
